# Standard Model Treatment of the Radiative Corrections to Neutron β-Decay

**DOI:** 10.6028/jres.110.047

**Published:** 2005-08-01

**Authors:** G. G. Bunatian

**Affiliations:** Joint Institute for Nuclear Research, 141980, Dubna, Russia

**Keywords:** neutron *β*-decay, radiative corrections

## Abstract

Starting with the Standard Model electroweak Lagrangian, the radiative corrections to neutron *β*-decay are obtained. Nucleon compositeness is considered by appropriate parameterization of the nucleon weak transition current and electromagnetic form factors.

## 1. Introduction and Discussion

The present treatment [1] of the neutron *β*-decay,

$$n\Rightarrow {\text{p+e}}^{-}+\bar{\nu }+\gamma ,$$ (1)

is based on the Standard Model electroweak Lagrangian [2–5]

$${ℒ}^{\text{EW}}\left(A_{\mu },Z_{\mu },W_{\mu }^{\pm },H,\psi_{f},e,M_{Z},M_{W},M_{H},m_{f},\xi \right),$$ (2)

which specifies the propagators of electromagnetic, *Z*-, *W* ^±^-boson, Higgs, and fermion fields, and the interactions between these fields. The quantities $e=\sqrt{4\pi \alpha }$, *M*_Z_, *M*_W_, *M*_H_, *m*_f_ are the unit of charge, masses of the Z-boson, W-boson, Higgs-boson, and fermions, respectively; the Feynman gauge *ξ* = 1 is chosen. In calculating the neutron *β*-decay amplitude in the one-loop approach, we leave out the effects of Higgs-fermion interactions, since they are of the order of the Higgs coupling to fermions ≈ *m*_f_/*M*_W_ [2–5]. Only the first generations of leptons (e,*ν*_e_) and quarks (*u*-, *d*-quarks) come into the consideration.

The transition amplitude ℳ of the process in Eq. (1), when calculated in the one-loop approach directly in terms of the bare fields and parameters, is UV-divergent, and renormalization is necessary. The multiplicative renormalization of the Lagrangian in Eq. (2) is performed amenably to the non-minimal on-mass-shell (OMS) renormalization scheme [3–5]. Upon calculating the radiative corrections with the fields, masses, and coupling constants renormalized within the OMS renormalization scheme, the UV divergencies occurring in the loop expansion (of propagators as well as *S*-matrix elements) are absorbed in the infinite parts of the renormalization constants. Also the finite parts of the radiative corrections are fixed. These lead to physically observable consequences.

As the nucleon is a composite system of strong interacting quarks, the amplitude ℳ of the process in Eq. (1) is determined by

$$ℳ\cdot i{\left(2\pi \right)}^{4}\delta \left(P_{n}-P_{p}-p_{e}-p_{\nu }-p_{\gamma }\right)=\langle \Phi_{0p}^{\mathrm{q+}}\left(P_{p},\sigma_{p}\right),{\phi_{e}}^{+}\left(p_{e},\sigma -e\right),A\left(p_{\gamma }\right)\left|S_{\mathrm{int}}\right|\Phi_{0n}^{q}\left(P_{n},\sigma_{n}\right),\phi_{\nu }\left(-p_{\nu },-\sigma_{e}\right)\rangle ,$$ (3)

$$\text{where}\;S_{\mathrm{int}}\equiv S_{\mathrm{int}}\left(\infty ,-\infty \right)=T\;\exp\left(i\int d^{4}x{ℒ}_{\mathrm{int}}\left(x\right)\right)$$ (4)

is dictated by the general Lagrangian ${ℒ}_{\mathrm{int}}\left(x\right)={ℒ}_{\mathrm{int}}^{\text{EW}}\left(x\right)+{ℒ}_{\text{str}}^{\text{qq}}\left(x\right)$, incorporating both electroweak and strong interactions. Here $\Phi_{0p}^{q}{,}_{0n}$, *ϕ*_e,v_, and *A* stand to describe the quark systems, electrons, neutrinos and *γ* rays. Nowadays, there seems no option but to allow for the effect of strong interactions by introducing the baryon weak and electromagnetic form factors. The Born amplitude ℳ^0^, represented by the first diagram in Eq. (6), is written in terms of the bare vertexes *Γ*^eνW^, *Γ*^npW^(*k*) and bare W-propagator *D*^W^(*k*), depicted by the point, blob, and thin wavy line. As the momentum transfer $k^{2}\ll M_{N}^{2}$, we actually deal with

$$\Gamma_{\alpha }^{\text{npW}}\left(0\right)=\frac{e\left|V_{\text{ud}}\right|}{2\sqrt{2s_{W}}}\gamma_{\alpha }\left(1-g_{A}\left(0\right)\gamma^{5}\right),D_{\alpha \beta }^{W}\left(0\right)=\frac{-g_{\alpha \beta }}{M_{W}^{2}},s_{W}^{2}=1-\frac{M_{W}^{2}}{M_{Z}^{2}},$$ (5)

and the electromagnetic form factors $f_{\alpha }^{pp}\left(0\right)=\gamma_{\alpha }$, $f_{\alpha }^{nn}\left(0\right)=0$. The corrected renormalized amplitude **ℳ** is presented in the one-loop approach by the set of diagrams.

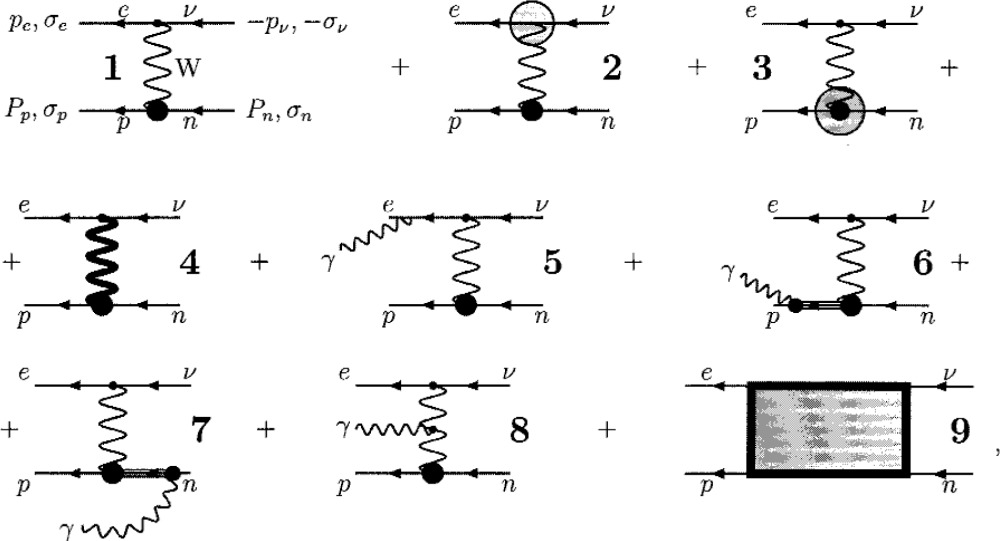
(6)

Calculation of the one-loop leptonic vertex ${\hat{\Gamma }}^{evW}$, depicted by the shaded circle in the diagram 2, is straightforward and results in merely a multiplicative factor to $\Gamma^{evW}$ Next, from *µ*-decay analysis [4], we obtain the corrected renormalized W-propagator ${\hat{D}}^{W}$, depicted by the heavy wavy line in the diagram 4, which replaces *D*^W^ in the diagram 1. As *m*, *M*_n_ − *M*_p_ ≪ *M*_p_ ≪ *M*_W_, the contributions from the diagrams 6 through 8 are negligible as compared to one coming out of the diagram 5, which renders the common bremsstrahlung of a final electron [6].

To treat strong interactions which are inherent in the processes described by the corrected renormalized vertex (the ${\hat{\Gamma }}^{\text{npW}}$ shaded circle with heavy core in the diagram 3), and by the irreducible four-fermion amplitude **ℳ**_2_*_γ_* (the “box-diagrams” 9), we split the virtual photon propagator, involved therein, into two parts

$$\begin{array}{l} D_{\mu \nu }^{A\lambda }\left(x_{2}-x_{3}\right)=g_{\mu \nu }\int \frac{d^{4}k}{{\left(2\pi \right)}^{4}}\left(\frac{1}{k^{2}-M_{S}^{2}+i0}+\frac{-M_{S}^{2}}{\left(k^{2}-\lambda^{2}+i0\right)\left(k^{2}-M_{S}^{2}+i0\right)}\right){\text{e}}^{-ik\left(x_{2}-x_{3}\right)}=\\ =D_{\mu \nu }^{As}\left(x_{2}-x_{3}\right)+D_{\mu \nu }^{Al}\left(x_{2}-x_{3}\right) \end{array}$$ (7)

where the subsidiary matching parameter $M_{S},M_{p}^{2}\ll M_{S}^{2}\ll M_{W}^{2}$ [2.7], emerges to separate large, $k^{2}\underset{˜}{>}M_{S}^{2}$, and comparatively small, $k^{2}\underset{˜}{<}M_{S}^{2}$, momenta transferred by the virtual photon. Then, the quantities ${\hat{\Gamma }}^{npW}$, **ℳ**_2_*_γ_* are divided into two parts incorporating these “massive” and “soft” photons with the propagators *D^As^*, *D^Al^*, respectively,

$${\hat{\Gamma }}_{\alpha }^{npW}={\hat{\Gamma }}_{s\alpha }^{npW}+{\hat{\Gamma }}_{l\alpha }^{npW},\;{ℳ}_{2\gamma }={ℳ}_{2\gamma s}+{ℳ}_{2\gamma l}.$$ (8)

In the quantities ${\hat{\Gamma }}_{s}^{npW}$, **ℳ**_2_*_γs_*, the electroweak interactions mediated by Z- and W-bosons and “massive” photons transfer the large momenta $k^{2}\underset{˜}{>}M_{S}^{2}$ to a quark system, so that strong quark-quark interactions die out, and quarks become asymptotically free in the respective intermediate states. Consequently, calculation of ${\hat{\Gamma }}_{s}^{npW}$, *M*_2_*_γs_* descends to evaluation of the transition matrix elements between the neutron $|\Phi_{n}^{q}\left(P_{n},\sigma_{n}\right)\rangle$ and proton $\langle \Phi_{p}^{q}\left(P_{p},\sigma_{p}\right)|$ states of the expressions given in terms of free quark operators:

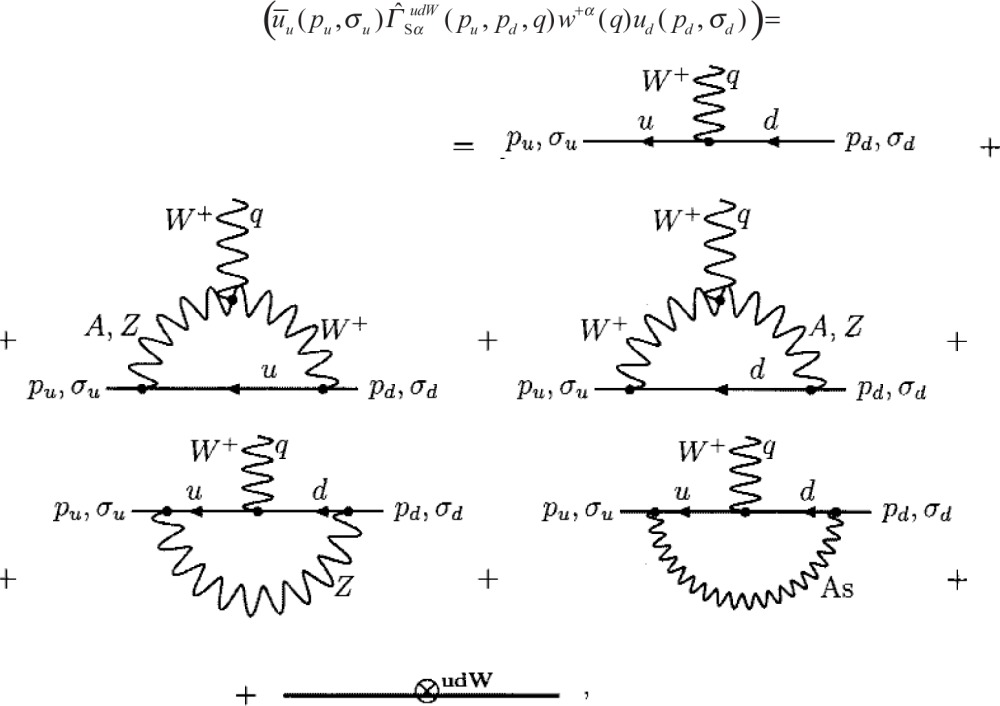
(9)in the case of ${\hat{\Gamma }}_{s}^{npW}$, $\left({\bar{\psi }}_{e}\left(x\right){\bar{\psi }}_{u}\left(x\right){\hat{\Gamma }}^{evud}\psi_{d}\left(x\right)\psi_{v}\left(x\right)\right)=$

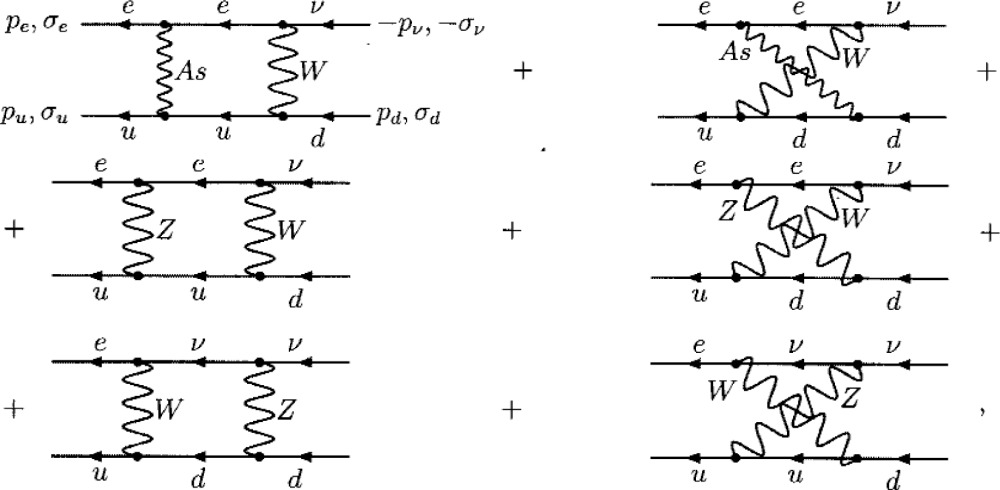
(10)in evaluating **ℳ**_2_*_γs_*. The wavy line with the tag *As* stands for the “massive photon” propagator *D_As_* from Eq. (7). Eventually, ${\hat{\Gamma }}_{s}^{npW}$, **ℳ**_2_*_γs_* prove merely to be proportional to Γ*^npW^*, **ℳ**^0^.

In the vertex ${\hat{\Gamma }}_{l}^{npW}$ and in the amplitude **ℳ**_2_*_γl_*, the “soft” photons transfer comparatively small momenta to a quark system, so that quarks constitute the baryon in such intermediate states. Actually, the prevailing parts of ${\hat{\Gamma }}_{l}^{npW}$, **ℳ**_2_*_γl_* are obtained by retaining only pure single nucleon intermediate states and presuming the vertexes and form factors found in Eq. (5). Then, ${\hat{\Gamma }}_{l\alpha }^{pnW}\approx \delta z^{p}\cdot \Gamma_{\alpha }^{npW}\left(0\right)/2$, where the UV-finite renormalization constant *δz^p^* of the proton wave function is defined in terms of the proton self-energy caused by “soft” photons of Eq. (7). The amplitude **ℳ**_2_*_γl_* results in

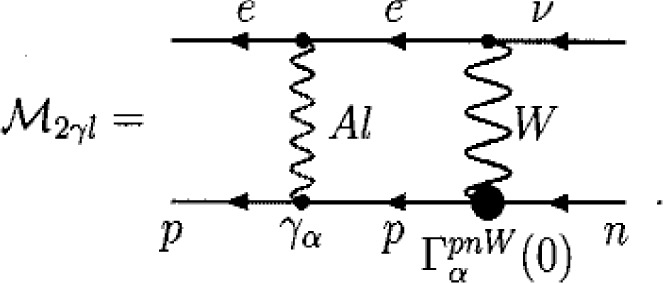
(11)The wavy line with the tag *Al* stands for the “soft” photon propagator *D^Al^* in Eq. (7). The quantity ℳ_2_*_γl_* of Eq. (11) turns out to be *not* a multiple of the Born amplitude ℳ^0^.

For now, strong interactions remain an unsatisfied calculational challenge. To realize the precision of the calculations, we size up how they are affected by allowance for (I) the contribution from the diagrams

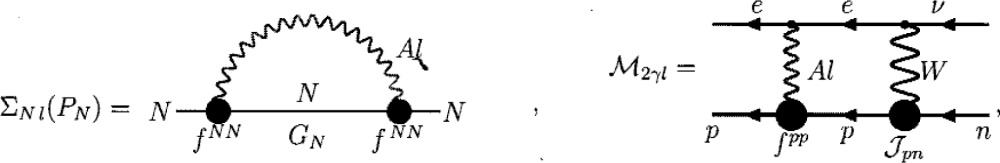
with the nucleon common transition currents and form factors

$$\begin{array}{c} J_{\text{np}}^{\beta }\left(k\right)=\gamma^{\beta }g_{v}\left(k^{2}\right)+g_{\text{WM}}\left(k^{2}\right)\sigma^{\beta \nu }k_{\nu }-\left(\gamma^{\beta }g_{A}\left(k^{2}\right)+g_{\text{IP}}\left(k^{2}\right)k^{\beta }\right)\gamma^{5},\\ f_{\alpha }^{NN}\left(k\right)=f_{1}^{NN}\left(k^{2}\right)\gamma_{\alpha }+f_{2}^{NN}\left(k^{2}\right)k^{\beta }\sigma_{\alpha \beta },\;g_{\text{WM}}\approx \frac{\mu_{n}-\mu_{p}}{2M_{p}}\approx \frac{3.7}{2M_{p}},g_{\text{IP}}\left(k^{2}\right)\sim \frac{8g_{A}\left(0\right)}{2M_{p}},\\ f_{1}^{pp}\left(k^{2}\right)\approx \frac{-m_{p}^{2}}{k^{2}-m_{p}^{2}},f_{2}^{pp}\left(k^{2}\right)\approx \frac{1.79}{2M_{p}}\frac{-m_{p}^{2}}{k^{2}-m_{p}^{2}}\;,f_{1}^{nn}=0\;,f_{2}^{nn}\left(k^{2}\right)\approx \frac{1.93}{2M_{p}}\frac{-m_{p}^{2}}{k^{2}-m_{p}^{2}}, \end{array}$$

and also (II) by allowance for insertion of the Δ_33_–isobar instead of the nucleon in the intermediate states in the diagrams

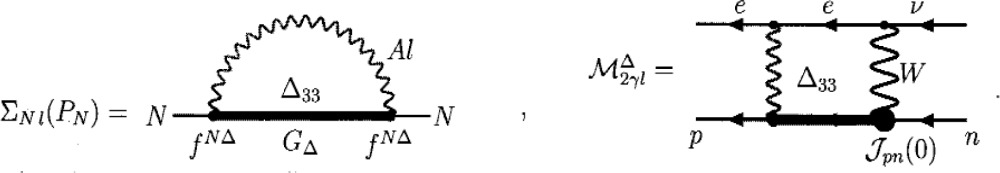


The thereby entailed effects prove to amount to about a few tenths of a percent, ≈ (0.1 – 0.3) %, to the value of decay amplitude ℳ. These estimations present the ambiguities inherent in the calculations. In fact, that is what restricts, together with the uncertainties ≈ 0.1 % due to the parameter *M*_S_ entanglement, the accuracy attainable in the present treatment of the neutron *β*-decay, without additional physical fit parameters, besides *g*_A_, |*V*_ud_|, involved.

With the amplitude ℳ from Eq.(6) as described above, the electron momentum distribution

$$d\text{W}\left(\varepsilon ,{\text{p}}_{e},\xi \right)=d\text{w}\left(\left(\varepsilon ,{\text{p}}_{e},\xi \right),{\text{p}}_{e}\right)\left\{W_{0}\left(g_{A},\varepsilon \right)+\upsilon \xi W_{\xi }\left(g_{A},\varepsilon \right)\right\}$$ (12)

turns out *not* to be a multiple of the quantity

$$\begin{array}{c} d{\text{W}}^{0}\left(\varepsilon ,{\text{p}}_{e},\xi \right)=d\text{w}\left(\varepsilon ,{\text{p}}_{e}\right)\left(1+3g_{A}^{2}+\upsilon \xi 2g_{A}\left(1-g_{A}\right)\right),\\ d\text{w}\left(\varepsilon ,{\text{p}}_{e}\right)=\frac{G^{2}{\left|V_{\text{ud}}\right|}^{2}}{2\pi^{3}}\varepsilon \left|{\text{p}}_{e}\right|k_{m}^{2}d\varepsilon \frac{d\text{n}}{4\pi },\text{n}={\text{p}}_{e}/\left|\text{p}\right|,\;\nu ={\text{p}}_{e}/\varepsilon ,k_{m}=M_{n}-M_{p}-\varepsilon , \end{array}$$ (13)

evaluated with the Born amplitude ℳ^0^, unlike what was asserted in the investigations of Refs. [8–10]. Let us note that Eq. (12) comprises all the *α*-order radiative corrections, without discarding the Coulomb term and separating the so called “model independent” and “model dependent” parts. Also, we nowhere appeal to the investigations of the 0^+^ → 0^+^ superallowed transitions in nuclei. If anything, introducing the new functions *λ*′(*ε*, ***p***_e_, *g*_A_), *λ*″(*ε*, ***p***_e_, *g*_A_), one might rewrite *W*_0_ = 1+3*λ*′^2^, *W_ξ_* = 2*λ*″(1 − *λ*″). Yet as *λ*′ ≠ *λ*″, it would be of no avail at all. The uncorrected asymmetry factor of the electron momentum distribution *A*_0_ is replaced by the quantity *A*(*ε*) accounting for the radiative corrections,

$$A_{0}=\frac{2g_{A}\left(1-g_{A}\right)}{1+3g_{A}^{2}}\Rightarrow \frac{W_{\xi }\left(g_{A},\varepsilon \right)}{W_{0}\left(g_{A},\varepsilon \right)}=A\left(\varepsilon ,g_{A}\right),\frac{A\left(\varepsilon ,g_{A}\right)-A_{0}}{A_{0}}=\delta A\left(\varepsilon \right)\approx -0.02\left(\pm \underset{˜}{<}0.002\right).$$ (14)

With *g*_A_ obtained, the radiative corrections cause the relative modification of the total decay probability *W*

$$\frac{\int_{m}^{M_{n}-M_{p}}d\varepsilon \;\varepsilon \left|{\text{p}}_{e}\right|k_{m}^{2}W_{0}\left(g_{A},\varepsilon \right)}{(1+3g_{A}^{2})\int_{m}^{M_{n}-M_{p}}d\varepsilon \;\varepsilon \left|{\text{p}}_{e}\right|k_{m}^{2}}-1=\delta W\approx 0.086\left(\pm \underset{˜}{<}0.003\right).$$ (15)

With the parameters obtained from [4, 5, 11], the CKM matrix element

$${\left|V_{\text{ud}}\right|}^{2}=\frac{5335}{\tau_{\exp}\left(1+3g_{A}^{2}\right)\left(1+\delta W\right)},$$ (16)

where *g*_A_ is determined by the *A*_exp_ value accordingly Eq. (14) and we directly arrive at

$$2g_{A}\left(1-g_{A}\right)=A_{\exp}\left(1-\delta A\right)\left(1+3g_{A}^{2}\right).$$ (17)

With the average values *τ*_exp_ = 885.7 s, *A*_exp_ = − 0.1162 from Ref. [11], we find *g*_A_ = 1.2729 and |*V*_ud_|^2^ = 0.9464. With *A*_exp_ = − 0.1189 ascertained in Ref. [12], the evaluation gives *g*_A_ = 1.2804 and |*V*_ud_|^2^ = 0.9372. It is to remark that presuming the value *τ*_exp_ = 878 s, reported in Ref. [13], we gain |*V*_ud_|^2^ = 0.9545 with the average value *τ*_exp_ = 885.7 s; with the values *τ*_exp_ = 878 s and *A*_exp_ = − 0.1189 from Refs. [12, 13], we arrive at |*V*_ud_|^2^ = 0.9453. As observed thereof, the deficiencies *∆* ≈ − 0.003—0.013 could be expected in the relation |*V*_ud_|^2^ + |*V*_us_|^2^ + |*V*_ud_|^2^ = 1 − *∆*, with the average values |*V*_us_|^2^ ≈ 0.0482 and |*V*_ub_|^2^ ≈ 2 · 10^−5^ from Ref. [11]. Considering these evaluations, we are to behold that their precision is about a few tenth of percent, $\underset{˜}{<}0.5\%$, as expounded above. So, strictly speaking, there seems no profound reason to assert an evidence of the CKM-unitarity violation, with accounting for the errors inherent in the *τ*_exp_ and *A*_exp_ values themselves as well [11–13].

Having at our disposal the amplitude ℳ of Eq. (6), we obtain [14] the modification of the recoil proton momentum distribution caused by the radiative corrections

$$\begin{array}{c} \delta \left(\left|P_{p}\right|,g_{A}\right)=\delta \left(\frac{dW_{p}\left(\left|P_{p}\right|,g_{A}\right)}{d\left|P_{p}\right|}\frac{1}{W_{p}\left(\left|P_{p}\right|,g_{A}\right)}\right)\approx \left(0.01-0.02\right),\\ \frac{dW_{\exp}\left(\left|P_{p}\right|\right)}{d\left|P_{p}\right|}\cdot \frac{1}{W_{\exp}\left(\left|P_{p}\right|\right)}=\frac{W_{0}\left(\left|P_{p}\right|,g_{A}\right)}{d\left|P_{p}\right|}\cdot \frac{1}{W_{p}\left(\left|P_{p}\right|,g_{A}\right)}\left(1+\delta \left(\left|P_{p}\right|,g_{A}\right)\right), \end{array}$$

which offers an additional condition to ascertain the *g*_A_ value from experimental data processing.

It should be noted that the final state of neutron *β*-decay given in Eq. (1) involves not three, but four particles because of the *γ* rays. Much needed high precision measurements of electron and proton momentum distributions will provide additional information on the quantities |*V*_ud_| and *g*_A_, yet not the neutrino correlation coefficients *B*, *a*, and *D* themselves [14–15].

Ingenious introduction of the redundant physical fit parameters, besides *g*_A_, |*V*_ud_|, to describe the effects of nucleon compositeness is believed to provide advancement in study the neutron *β*-decay.
